# Assessment of detraining through a six-minute walk test in patients with heart disease

**DOI:** 10.1590/1516-3180.2023.0334.R1.03072024

**Published:** 2024-12-20

**Authors:** Victória Moreira Silva, Vitória Moreira Cintra, Maria de Lourdes da Silva, Joilson Meneguci, Fernando Seiji Silva, Eduardo Elias Vieira de Carvalho, Ana Paula Espindula, Lucina Duarte Novais Silva

**Affiliations:** I Graduate Student, Department of Applied Physical Therapy, Universidade Federal do Triângulo Mineiro (UFTM), Uberaba (MG), Brazil.; II Department of Applied Physical Therapy, Universidade Federal do Triângulo Mineiro (UFTM), Uberaba (MG), Brazil.; III Graduate student, Department of Applied Physical Therapy, Universidade Federal do Triângulo Mineiro (UFTM), Uberaba (MG), Brazil.; IV Teaching and Research Management, Postgraduate Program in Physical Education, Universidade Federal do Triângulo Mineiro (UFTM), Uberaba (MG), Brazil.; V Professor, Department of Structural Biology, Universidade Federal do Triângulo Mineiro (UFTM), Uberaba (MG), Brazil.; VI Professor, Department of Applied Physical Therapy, Universidade Federal do Triângulo Mineiro (UFTM), Uberaba (MG), Brazil.; VII Researcher, Department of Structural Biology, Universidade Federal do Triângulo Mineiro (UFTM), Uberaba (MG), Brazil.; VIII Professor, Department of Applied Physical Therapy, Universidade Federal do Triângulo Mineiro (UFTM), Uberaba (MG), Brazil.

**Keywords:** Walk test, Heart diseases, Cardiac rehabilitation, Exercise, Physical fitness, Principle of reversibility, Cardiovascular rehabilitation program, Six-minute walk test, Physical capacity, Exercise test, Cardiovascular diseases

## Abstract

**BACKGROUND:**

Detraining can partially or completely reduce training-induced metabolic adaptations. However, the duration for which the rehabilitation effects persist after detraining, especially in patients with heart disease, remains unclear.

**OBJECTIVES:**

To evaluate the principle of reversibility/detraining in patients with heart disease via the 6-minute walk test (6MWT) after a period of rest.

**DESIGN AND SETTING:**

A retrospective cohort study developed at the Rehabilitation Center of the Universidade Federal do Triângulo Mineiro in Uberaba/MG, Brazil.

**METHODS:**

This clinical, retrospective longitudinal study involved 20 patients with heart disease who underwent 5 months of supervised cardiac rehabilitation (CR). The mean age of participants was 64.05 ± 9.25 years. The initial rehabilitation was followed by an interruption period and rehabilitation for another 5 months. Functional capacity was assessed using the 6MWT.

**RESULTS:**

In the specific analysis of the distance covered, values of P = 0.03 and P = 0.01 were obtained on comparing post-training (669.64 ± 58.80 meters) with post-detraining (640.82 ± 101.23 meters) and post-detraining with post-retraining (650.82 ± 96.28 meters), respectively. No significant difference was observed for the comparison between training and retraining (P = 0.83).

**CONCLUSION:**

Cardiovascular rehabilitation positively stimulates functional capacity, whereas detraining significantly reduces it. The 6MWT is effective in measuring changes in physical capacity.

## INTRODUCTION

Cardiovascular diseases (CVDs) are the leading cause of mortality worldwide, accounting for a 21.1% surge in total deaths from 2007 to 2017.^1,2^ Primary prevention strategies for CVDs involve lifestyle modification (e.g., smoking cessation and increased physical activity) and drug therapy.^3^

Epidemiological findings have demonstrated an association between physical inactivity and a higher prevalence of most CVD risk factors, including dyslipidemia, high blood pressure (BP), metabolic syndrome, obesity, and type 2 diabetes.^4^

Cardiovascular rehabilitation (CR) is a chronic disease management program that provides structured exercises, patient education to promote behavioral changes, and psychological support to lessen the burden of risk factors and optimize secondary prevention.^5^ Notably, CR increases functional capacity with continued gains over the following months.^6^

Continuous rehabilitation through physical exercise is vital for patients with heart disease to reduce limitations and ensure a better quality of life. The suspension or reduction of this training can cause deconditioning, affect performance, and decrease physical capacity.^7^

This interruption, called the principle of reversibility or detraining (DT), can occur due to an injury, aging, voluntary discontinuation, and/or as part of the annual training cycle to train for other skills.^8^

DT can be assessed through the 6-minute walk test (6MWT). Notably, disease progression and the risk of hospitalization or mortality can be assessed by measuring the distance covered,^9,10^ and the variables measured in the 6MWT exhibit a strong correlation with the cardiopulmonary test, which is the gold standard for assessing physical capacity.^11^ Although many studies have examined the effects of CR in patients with heart disease, the duration for which the rehabilitation effects persist after the DT stage remains unclear. In addition, studies on DT have been mainly conducted on the athlete population than the non-athlete population.^12^

Therefore, more studies, especially involving patients with heart disease, considering both adherence and the importance of continuing physical training to chronically maintain the benefits achieved via training, are necessary. DT may lead to a partial or complete reduction in training-induced physiological adaptations and performance.

## OBJECTIVES

The present study primarily aimed to assess the principle of reversibility/detraining via the 6MWT in patients with heart disease undergoing CR after a period without training. We also reassessed the patients after a new training period.

## METHODS

This retrospective study involved patients assisted at a rehabilitation center. This center operates in different areas, including physiotherapy rehabilitation, medical screening consultations, occupational therapy, nutrition, nursing, and psychology. The participants were selected from a convenience sample of 20 patients who underwent CR.

Patients with CVDs of both sexes, aged > 18 years, who regularly participated in our institution’s CR program for at least 5 consecutive months were included in the study. Patients who did not undergo the 6MWT and those with a participation frequency of < 80% were excluded from the study.

The patients included in this retrospective analysis followed the institutional protocol of the CR program, as follows: initially, all program patients were evaluated by a cardiologist specializing in ergometry and rehabilitation who, following a thorough clinical assessment, including the review of complementary examinations and medication optimization, when necessary, performed a conventional limited physical exertion ergometric test. This test was conducted by a cardiologist before the patient was enrolled in the rehabilitation program to assess signs and symptoms during physical exertion, cardiovascular risk classification, and the initial prescription of physical training intensity based on heart rate (HR). Subsequently, all patients included in our rehabilitation program underwent follow-up re-evaluations through the 6MWT whenever they were about to interrupt the program for any reason or were progressing to the next phase.

All patients were explained about the study, and signed informed consent was obtained from all participants with consent to allow the use of the results of their evaluations. The study was approved by the Research Ethics Committee of the Universidade Federal do Triângulo Mineiro (UFTM) (protocol 3.378.424; 06/07/2019).

### Evaluation Period

Three evaluations were performed, as follows:

- First evaluation (post-training): At the end of 5 months of CR (n = 20).
- Second evaluation (post-DT): After 45 days of interruption, with no loss of the sample (n = 20).
- Third assessment (post-retraining): At the end of the 5-month rehabilitation period (retraining period) involving individuals who were able to attend and were actively attending the program (n = 11) (**Figure 1**). Of the 20 patients, 9 were excluded from the study because they did not adhere to the treatment or did not show up for evaluation.

Therefore, the study was divided into two phases. The first phase involved the assessment of the effect of detraining on the physical capacity of the patient, measured by comparing the distance covered in the post-training and post-DT tests after the second evaluation (n = 20). The second phase involved comparing the distance covered at three time points: post-training, post-DT, and post-retraining after the third evaluation (n = 11).

### Study Protocol

#### Cardiovascular rehabilitation

The CR lasted 60 minutes, as follows: a warm-up characterized by stretching large muscle groups (10 minutes); aerobic conditioning in ergometers, such as a treadmill or bicycle (30 minutes); peripheral muscular resistance training of the upper and lower limbs using dumbbells and anklets (10 minutes); and ending with a cool-down (10 minutes). The conditioning intensity was determined through an ergometric test, with 60–80% of the reserve HR of the maximum HR reached in the test calculated using the Karvonen method. This test was performed routinely at the rehabilitation center for all individuals prior to the start of rehabilitation.

During the 45-day interruption of the CR program, patients were advised to maintain their usual activities and were counseled regarding the benefits of physical training. However, unsupervised training was not prescribed during this period.

### Six-minute walk test

The 6MWT was always performed in the morning, and patient information, including registration number, sex, height and weight (for calculating the body mass index [BMI]), age, waist circumference, BP, and HR, was collected.

Data were collected from the medical records of the 6MWT, which is routinely performed at our rehabilitation center, prioritizing the same evaluator to guarantee the reliability of the test. The test was performed in accordance with the guidelines of the American Thoracic Society,^13^ requiring a corridor with a minimum length of 30 m free of human circulation, two cones to delimit the route, and a stopwatch to record the time. The participants were instructed to walk on the track as quickly as possible for 6 minutes. Before performing the test, the patients rested for at least 10 minutes, after which resting BP was evaluated using a Bic aneroid sphygmomanometer and Littmann Classic 3m stethoscope, and resting HR was evaluated using a Polar heart rate monitor, model FS2. The patients were given instructions on how to perform the test.

The patients underwent two tests with a minimum interval of 15 minutes. Notably, the test with the greatest distance covered in 6 minutes was considered for rest and normalization of vital data. After performing the test, the distance walked, defined as the maximum distance that the patient was able to cover during the test, was evaluated, along with the level of effort using the Borg Scale, BP of recovery, and HR of recovery immediately after completion. Notably, two measurements were taken at 2 and 4 minutes after the test was interrupted, and the predicted distance covered by each patient was calculated.^14^

### Statistical Analysis

Statistical analyses were performed using SPSS 19.0 (IBM, Armonk, New York, United States). Descriptive analysis was performed by calculating the central tendency (mean and median) and dispersion (standard deviation [SD]). The Shapiro–Wilk test was used to verify the normality of the data. Paired Student’s t-test and Wilcoxon test were used for variables with normal and non-normal distributions, respectively. The Friedman test with multiple comparisons of the averages of orders was used to compare the variables at specific time points (post-training, post-DT, and post-retraining). The significance level was set at 5%.

## RESULTS

Twenty individuals who underwent CR participated in this study. The mean age was 64.1 ± 9.3 years, with a prevalence of 70% and 30% in males (n = 14) and females (n = 6), respectively. The most frequent comorbidity was systolic hypertension (70%, n = 14), followed by acute myocardial infarction (45%, n = 9). Notably, only 5% (n = 1) of the participants were smokers, and 25% (n = 5) have smoked in the past (**Table 1**).

**Table 1:** Patient characteristics (n = 20)

Variables	P value
Age, years	64.1 ± 9.3
**Sex**	n (%)
Female	6 (30.0)
Male	14 (70.0)
**Comorbidities/intervention**	
Hypertension	14 (70.0)
Acute myocardial infarction	10 (50.0)
Coronary transluminal angioplasty	10 (50.0)
Cardiomyopathy	7 (35.0)
Diabetes mellitus	6 (30.0)
Dyslipidemia	6 (30.0)
Familial hypercholesterolemia	5 (25.0)
Previous smoking	5 (25.0)
Myocardial revascularization surgery	3 (15.0)
Arterial fibrillation	2 (10.0)
Obesity	2 (10.0)
Heart valve diseases	2 (10.0)
Angina	2 (10.0)
Hypothyroidism	1 (5.0)
Left ventricular aneurysm	1 (5.0)
Infective endocarditis	1 (5.0)
Metabolic syndrome	1 (5.0)
Smoker	1 (5.0)
Physical activity on vacation	6 (30.0)

No sample loss occurred between the post-training and post-detraining periods. A significant reduction was observed in HR at rest (P = 0.04) and distance covered (P = 0.02; **Table 2**).

**Table 2:** Comparison of variables between post-training and post-DT (n = 20)

Variables	Post-training	Post-DT	P value
BMI (Kg/m^2^)	28.3 ± 5.5	28.4 ± 5.6	0.69
Abdominal circumference (cm)	98.4 ± 11.0	94.55 ± 22.95	0.76
SP rest (mmHg)	121. 5 ± 15.0	111.5 ± 16.3	0.07
SP recovery (mmHg)	138.5 ± 32.2	138.5 ± 25.4	1
DP rest (mmHg)	84.5 ± 15.4	83.0 ± 10.3	0.95
DP recovery (mmHg)	84.0 ± 9.9	85.0 ± 9.5	0.52
HR rest (bpm)	72.7 ± 11.8	68.6 ± 9.1	0.04*
HR recovery (bpm)	108.7 ± 18.1	107.7 ± 19.7	0.71
Distance covered (m)	618.9 ± 94.9	583.6 ± 120.4	0.02*
Borg effort scale	2.5 ± 0.9	2.8 ± 0.9	0.08
Predicted distance covered (m)	505.8 ± 75	--	

SD = standard deviation; BMI = body mass index; SP = systolic pressure; DP = diastolic pressure; HR = heart rate; rest = evaluated before the test; recovery = evaluated at the end of the test;

*P < 0.05.

During retraining, 9 patients were dropped out owing to their absence from rehabilitation, and 11 patients finally participated in all stages of data collection. A significant difference was only observed in the distance covered when comparing the results of the post-training, post-DT, and post-retraining periods (n = 11, Friedman test, P = 0.03; **Table 3**). Comparisons of the training period with post-DT and post-DT with post-retraining exhibited P = 0.03 and P = 0.01, respectively, in the specific analysis of the distance covered. No significant difference was observed between the post-training and post-retraining periods (P = 0.83).

**Table 3:** Comparison of variables among post-training, post-DT, and post-retraining (n = 11)

Variables	Post-training	Post-DT	Post-retraining	P-value
BMI (Kg/m^2^)	27.2 ± 2.9	27.1 ± 2.9	27.4 ± 2.6	0.67
Abdominal circumference (cm)	95.4 ± 5.8	89.3 ± 27.4	94.0 ± 6.0	0.35
SP rest (mmHg)	122.7 ± 15.6	115.5 ± 12.9	120.9 ± 19.7	0.15
SP recovery (mmHg)	137.3 ± 37.2	140.0 ± 29.7	140.5 ± 36.1	0.64
DP rest (mmHg)	89.1 ± 18.1	84.6 ± 5.2	79.1 ± 8.3	0.12
DP recovery (mmHg)	86.4 ± 8.1	88.2 ± 4.0	80.0 ± 12.6	0.07
HR rest (bpm)	71.6 ± 13.4	65.7 ± 9.2	73.1 ± 11.2	0.21
HR recovery (bpm)	109.5 ± 20.4	109.0 ± 19.1	112.0 ± 25.0	0.92
Distance covered (m)	669.6 ± 58.8*	640.8 ± 101.2	650.8 ± 96.3^#^	**< 0.05**
Borg effort scale	2.4 ± 0.8	2.6 ± 0.8	2.1 ± 1.4	0.33

SD = standard deviation; BMI = body mass index; SP = systolic pressure; DP = diastolic pressure; HR = heart rate; rest = evaluated before the test; recovery = evaluated at the end of the test; Friedman test

*P = 0.03 compared withpost-DT, and ^#^P = 0.01 in compared withpost-DT.

## DISCUSSION

The results of the present study revealed that the distance covered in the 6MWT was an effective parameter for documenting the reduction in physical capacity after a 45-day period of interruption of supervised training in patients with CVD. Additionally, the individuals reattained the pre-interruption training parameter values after a period of 5 months of retraining.

Regular exposure to long-term exercise (physical training) promotes a set of morphological and functional adaptations involving intrinsic and extrinsic mechanisms of the heart, thus increasing the capacity of the body to respond to exercise stress.^15^ Furthermore, regular physical training can promote various adaptations, including the growth, proliferation, and function of cardiomyocytes; mitochondrial biogenesis; improved lipid and glucose metabolism; changes in the morphology and function of the microcirculation; prevention of cardiac fibrosis; control of systemic and cardiac inflammation; beneficial changes in the microbiome; and positive adaptations in all body systems.^16^ Conversely, an interruption or reduction of this training leads to a deconditioning process, with a reduction in the gains obtained.^7^

According to the World Health Organization,^17^ approximately 600 million people exhibit high BP, which may increase by 60% by 2025. Notably, this is consistent with the results of the present study, wherein the most frequent comorbidity was systolic arterial hypertension (70%, n = 14). According to the 2017 Surveillance System of Risk and Protective Factors for Chronic Diseases by Telephone Survey, the prevalence of self-reported hypertension increased from 22.6% in 2006 to 24.3% in 2017.^18^ Notably, reducing salt intake and encouraging physical activity and healthy eating are the most effective measures to reduce BP.^19^

Our findings revealed a significant decrease (P = 0.02) in the distance covered after the initial detraining (from 618.9 ± 94.9 meters to 583.6 ± 120.4 meters), indicating a loss in the functional capacity of the individual acquired through CR. This result corroborates the findings of the study by Seemann et al.,^20^ involving the assessment of the influence of detraining in older women who underwent a functional gymnastics program, which reported a decrease in performance, as evaluated through the 6MWT, after 3 months of interruption in the functional gymnastics sessions.

Detraining occurs quickly, even after a few weeks of interruption, along with significant reductions in the ability to perform work and almost total loss after a few months.^21^ Additionally, some factors, such as the decline in cardiac output, arteriovenous difference, oxidative enzymes, hemoglobin concentration, density of myocytic mitochondria, and muscle capillarization, occurring alongside detraining may contribute to the reduction in aerobic fitness.^22,23^

According to Sousa et al.,^24^ 8 weeks of aerobic and resistance training are sufficient to verify improvements, whereas only 4 weeks of detraining results in a marked reduction in physical performance, with the variables returning to their initial values. These results align with those of other studies, which reported that training-induced gains can be compromised with a short-term detraining period (6 weeks), leading to a decrease in performance and return to baseline values.^25,26^ This information can be useful for health professionals to encourage individuals not to interrupt training, as the acquired capacities are lost and interfere with their physical fitness.

Despite detraining, the patients covered a good distance in the 6MWT. The average predicted distance covered by the patients was 505.8 ± 75 m, with the distance covered in the post-training test being 125.5% higher than predicted. Similarly, the distance covered in the post-DT test was also higher than predicted (115.5%), despite significantly lower values than post-training.

This result highlights that a 45-day period without supervised physical training is sufficient to significantly reduce physical capacity in patients with CVD participating in a CR program. However, patients could still cover a good distance (greater than the predicted distance) in 6MWT.

In this study, we calculate the predicted distances using the formula described by Enright and Sherrill.^14^ However, other formulae are also available in the literature. Iwama et al.,^27^ in their study involving 134 healthy Brazilians of both sexes, aged between 13 and 84 years, described an equation to predict the distance covered and documented the existence of variances among the other formulas described in the literature.

However, we used the equation by Enright and Sherrill^14^ in our study based on another study that tested this formula in a population of Brazilian adults with cardiac comorbidities. Moreover, Costa et al.^28^ documented a significant correlation between the actual distance covered and the distance estimated using this equation.

In the present study, no significant difference was observed (P = 0.83) on comparing the distance walked after the training period (669.6 ± 58.8 meters) with the distance walked at the end of the retraining (650.8 ± 96.3 meters). Although the results did not return to the initial values, the functional capacity lost during the detraining period was recovered after 5 months of training.

Lee et al.,^29^ on comparing functional fitness in the older individuals 12 months after training, 12 months after detraining, and 3, 6, and 9 months after retraining, found improvement in aerobic resistance post-training, whereas functional fitness of the participants decreased by 16.59% post-detraining. Notably, a refreshment period of ≥ 9 months was necessary to gradually recover the post-training condition. A study by Tokmakidis et al.,^30^ involving combined training (aerobic and strength) for 9 months, followed by 3 months of detraining and 9 months of retraining, revealed that the discontinuation of exercise also led to negative changes; however, all beneficial adaptations were restored during the retraining period.

In the present study, functional capacity in patients with CVD was increased after 5 months of rehabilitation (P = 0.01), as evidenced by an increase in the distance covered post-DT (640.8 ± 101.2 meters) than post-retraining (650.8 ± 96.3 meters). These findings align with the findings of Silva et al.,^31^ who compared the functional capacity of patients with cardiac disease before and after a 60-minute training for 8 weeks, totaling 16 sessions. Based on the 6MWT results, conventional CR positively influenced the physical capacity of individuals.

The study of Lima et al.,^32^ investigating the effects of a 12-week aerobic training program involving moderate-intensity aerobic activities (warm-up, stretching, walking, stretching, and relaxing) for 50 minutes on the functional capacity of sedentary women with hypertension during the menopausal period, revealed an increase in the distance covered in the 6MWT after the practice period. Therefore, aerobic training may improve functional capacity. Bustamante et al.,^33^ through a survey including 277 individuals who were integrated into phase II of CR, which lasted 36 sessions (approximately 12 weeks), revealed a significant improvement of 10% (mean, 56.4 meters) in the distance covered in the 6MWT.

Although physical training improves maximal aerobic power, it does not significantly modify maximal HR. Notably, patients undergoing aerobic training achieve the same maximum HR as before training, but more intense efforts are required to reach maximum HR.^34^

Evaluating the effects of detraining is challenging, and consensus remains lacking in the literature owing to different types of training with different intensities, frequencies, and durations. Notably, divergent findings have been reported from similar studies, indicating the existence of many variables that can influence the final result. Consequently, further studies evaluating different detraining times in patients with cardiac disease are necessary to better understand the changes in functional capacity in this population.

The results of the present study demonstrate that the 6MWT, a simple and low-cost tool, is effective for training and detraining assessments. Moreover, as the 6MWT has shown a strong correlation with cardiopulmonary exercise testing,^11,35^ which is considered the gold standard for assessing physical capacity, the 6MWT becomes an effective tool for serial evaluations. Notably, a small sample (n = 20) is one limitation of this study, with a more pronounced decrease in the sample size following the loss of nine participants over the 5-month retraining period.

## CONCLUSION

Detraining reduced the training-induced gains in the study participants, negatively influencing the functional capacity of patients with CVD. The 6MWT was effective in measuring changes in physical capacity.
